# Factors explaining the fear of being infected with COVID‐19

**DOI:** 10.1111/hex.13274

**Published:** 2021-05-11

**Authors:** Arcadio A. Cerda, Leidy Y. García

**Affiliations:** ^1^ Faculty of Economics and Business University of Talca Talca Chile; ^2^ Faculty of Economics and Business University of Talca Talca Chile

**Keywords:** Chile, health care, pandemic, perception, probit model

## Abstract

**Background:**

The prevalence of COVID‐19 has a social and economic impact on people, leaving them distressed and fearful of getting infected.

**Objective:**

To determine the variables attributable to the fear of contracting COVID‐19.

**Design:**

This is a quantitative study based on an online cross‐sectional self‐administered survey in Chile between 10 July 2020 and 10 August 2020.

**Setting and participants:**

A sample of 531, comprising over 18‐year‐old participants from middle‐ and high‐income levels, was selected.

**Outcome measures:**

Estimations were obtained using a probit regression model with marginal effects.

**Results:**

Fear prevailed mainly in women. It has a positive relationship with variables such as chronic illnesses, infectious family or relatives, reduction in economic activity and perception of bad government response to a pandemic. Fear has a negative relationship with knowledge about COVID‐19, education level and ageing. Moreover, those who consider socioeconomic impact less important than health care do not fear a COVID‐19 infection.

**Discussionand conclusion:**

The socioeconomic and health aspects help predict fears. Thus, the government should prioritize these variables in implementing policies. The government's credibility and communication systems can also reduce fears of contracting COVID‐19.

**Patient or public contribution:**

A pilot focus group of COVID‐19–recuperated individuals and some members of our interest groups were consulted in the design stage of the study; this helped in constructing the survey questions. Additionally, three independent individuals volunteered to read and comment on the draft manuscript.

## 1 INTRODUCTION

The duration of the pandemic has undoubtedly affected the behaviour of people and has triggered fear in many people, because people perceive an increase in the risk of getting sick, especially after the number of infected people increased or a family member became ill or died from being infected by COVID‐19.^1^, ^2^ Fear is defined as an unpleasant emotional state caused by the perception of menacing stimulus.^3^ Therefore, the longer the pandemic lasts, the greater the fears that people may face, affecting their socioeconomic well‐being^4^ and mental health.^5^, ^6^, ^7^, ^8^, ^9^ However, in some cases, the longer it takes to find a solution to the pandemic, the more people will not be inclined to take adequate measures to avoid contagious behaviour (social distancing, washing hands and quarantine compliance), because they are motivated to act not only for health‐care issues but also for social and economic reasons (income generation, human relationships or simply contempt with authority). This situation is evident when we observe the second and third pandemic wave around many countries in the globe where people have not received enough health care.

Comparing the pandemic situation a month and a half after the first case of COVID‐19 was diagnosed in Chile (17 April 2020) with the existing situation when we realized this study (19 July 2020), the cases and deaths have increased by 3 176% (3 030 083) and 5 566% (6573), respectively,^10^ while the cases and deaths have increased by 4.35% and 2.60%, respectively, for the same period.^11^ According to the Central Bank of Chile, the monthly Economic Activity Index between June 2020 and 2019 fell by 12.4%.^12^ The number of unemployed people increased by 42.9%, which is the highest unemployment level in Chile between 2010 and 2020.^13^ Moreover, from 7 July 2020 to 4 February 2021, the number of deaths and infected people has increased significantly. Specifically, the number of infected people has increased by 144%, reaching a total of 740 237 people, and the number of deaths increased by 185%, reaching a total of 18 731.

Aspects such as restrictive movement policies, confinement to residential properties, saturation of the health‐care system, quality of government response, loss of employment, financial loss, number of infected people, trust in people and/or institutions are some of the factors that may strongly influence people's well‐being and their fears of contracting the disease.^14^ All these factors became relevant when there was no certainty about a vaccine and its characteristics. The media coverage may continue to provide information about contagious and perished people.^15^, ^16^, ^17^

The health economics literature is primarily concerned with determining the willingness to pay for a vaccine and determining individuals' preferences for a vaccine under different modalities of effectiveness and side‐effects. The preferences and the willingness to pay for a vaccine could express an individual's fears of getting infected.^1^, ^8^, ^18^, ^19^, ^20^, ^21^

Torales, O'Higgins, Castaldelli‐Maia and Ventriglio^5^ mentioned that the pandemic generates stress, anxiety, depression, insomnia, anger and fear globally. For example, home confinement causes a negative psychological impact, affecting the emotional state because of depression and anxiety.^22^ The authors added that collective concerns could affect individual daily behaviours, reducing the effectiveness of COVID‐19 strategies and control. Debiec and Olsson^23^ mentioned that fear responses could be learned, directly or indirectly, by social transmission. Thus, fear contagion may often happen automatically and unconsciously, making it complicated to control.^24^ This can be even more difficult to manage as the number of cases of COVID‐19 increases, and the media provides information about infected and dead people.

However, governments sometimes instil fear in people to raise health awareness of COVID‐19, to change intentions and to motivate them to be vigilant during the pandemic.^15^, ^16^, ^22^ In addition, government's response and credibility can affect the acceptability of vaccination.^25^, ^26^ As mentioned by Jakovljevic, Sugahara, Timofeyev and Rancic,^27^ the quality of governance determines health‐care system performance. However, vaccine rejection may decrease depending on the severity of COVID‐19 infections. It can also decrease if people are aware of vaccination benefits or have family members infected with the virus. ^1^, ^2^

Clark, Davila, Regis and Kraus^28^ show that trust in the government has a low influence on individual decisions to take other preventive actions (mask‐wearing, social distancing, hand washing and staying at home) to reduce the probability of getting infected with COVID‐19. Mertens, Gerritsen, Duijndam, Salemink and Engelhard^29^ found that fear is caused by health anxiety, regular media use, social media use and risk for loved ones. People's fear is triggered not only by COVID‐19 infections but also by the consequences of the pandemic, such as employment loss, income loss and affected social relationships.^1^, ^30^ For example, according to Pakpour and Griffiths,^15^ people from both low‐income and middle‐income countries are worried about their jobs.

Few studies have explored the main factors that explain the fears of people during the current pandemic. This study aimed to analyse the main factors influencing people's fear of being infected with COVID‐19 to implement appropriate policies aimed at reducing people's fears during the COVID‐19. These factors will be helpful even in future occurrences of pandemics. As mentioned by Li, Yue and Crabbe,^31^ the appearance of SARS in 2003 and COVID‐19 in 2019 indicates that it is possible that other coronavirus diseases may occur again in the future.

## 2 MATERIALS AND METHODS

### 2.1 Study design

The data were collected through an online self‐administered questionnaire distributed to various social media platforms between 10 July 2020 and 10 August 2020. The sample comprised 531 Chilean participants aged between 18 and 90 years, mainly in the middle‐ and higher income levels. The questionnaire was validated by experts and other select individuals through a focus group and a pilot survey.

Furthermore, we asked for the opinions of a small focus group of COVID‐19 survivors and some members of our interest group in the design stage of the study. This helped to refine the survey questions regarding fear issues. Additionally, three independent individuals volunteered to read and comment on the drafted manuscript.

### 2.2 Sample

We considered a sample of 531 respondents, resulting in a margin error of 4.25% and a confidence level of 95%, assuming maximum variance and infinite population. Among these participants, 59% were men, and 41% were women; 52.0% of all of them had a private health‐care system. Most respondents belong to middle‐income and high‐income levels (83%), and only a few belong to the low‐income (17%) group. Almost 77% are graduates.

The questionnaire included three main parts: the first part had 20 questions related to knowledge about the pandemic, individuals’ perception and fear of their risk of infection, economic and social impacts, and information on medical conditions (chronic diseases, if any). We used the Likert scale with 1 denoting ‘completely disagree’ and 5 denoting ‘completely agree’ or dichotomous questions (yes or not).

The second part of the questionnaire had questions about the willingness to pay for vaccines and reasons for rejecting them. Finally, the third part had ten questions about sociodemographic data and children's education and performance under the pandemic. However, the data obtained from the second and third part questions about willingness to pay and child education were not considered in this study. However, it has been used for other publications, where the questionnaire is available as supplementary material on Cerda et al. ^1^

We notified the respondents that there is no monetary compensation, the survey is voluntary, and they could stop participating at any time. They were also informed that the data would be generalized: no. individual data and no record of their identities will be revealed. All participants consented to partake in the study.

### 2.3 Estimation output method

The estimation method consisted of a probit model, expressed as:

$$y_{i}\left\{\begin{array}{c} \text{1 the level of fear has increased}\\ \text{0 the level of fear has not increased} \end{array}\right.\forall_{i}=\text{1, 2,}\ldots ,n.$$

where the standardized normal cumulative distribution probability function is represented as:

$$\text{Pr}\left(y_{i}=\frac{1}{x_{i}}\right)=\int_{‐\infty }^{x_{i}\beta }\frac{1}{\sqrt{2\pi }}\exp\left[‐\frac{t^{2}}{2}\right]dt,$$

where *x_i_* represents the matrix of explanatory variables for the individual *i*, *β* represents the vector of coefficients, and *t* denotes the normal standardized distribution. The estimation of the parameters was carried out through the maximum‐likelihood method. The estimated marginal effect of an independent variable *x* is the partial derivative, with respect to *x*, of the prediction function. The model was estimated using the statistical software STATA 16.0.

## 3 RESULTS

### 3.1 Data analysis

The data show that 290 respondents (54.6%) of the 531 individuals showed more fear of contracting the virus after the first and a half months since the pandemic hit Chile. Women's fear surpassed that of men. Specifically, women's fear increased by 89%, while fear of men increased by 30%.

Comparing middle‐aged adults (30 to 59 years) with younger adults (19 to 29 years) and senior people (older than 60 years) yielded the same results to that of women and men proportions. Additionally, people from a lower‐income (64.6%) group had more fears than those from other income‐level groups. Respondents with a higher level of education (45.8%) had less fear than those with a lower level of education (54.2%) (Table 1).

**Table 1:** General sample description

Characteristics	Sample description n (%) 531 (100) (1)	Number of people who responded that they had increased their fears of getting infected (%) 290 (100) (2)	Percentage of respondents with more fears over the total value of each characteristic (%) (3)
Age			
18‐29	61 (11.5)	33 (11.4)	54.1
30‐39	120 (22.6)	70 (24.1)	58.3
40‐49	136 (25.6)	73 (25.2)	53.7
50‐59	130 (24.7)	75 (27.9)	53.9
60 and more	83 (15.6)	39 (13.4)	47.0
Gender			
Female	212 (39.9)	177 (61.1)	83.5
Male	314 (59.3)	110 (37.9)	35.0
No response	4 (0.8)	3 (1.0)	75.0
Education			
Preliminary school	6 (1.1)	3 (1.0)	50.0
High school	45 (8.5)	30 (10.3)	66.7
Technical	73 (13.8)	45 (15.5)	61.6
Undergraduate degree	231 (43.7)	132 (45.5)	57.1
Graduate degree	175 (33.0)	80 (27.6)	45.7
Monthly income (US dollars)			
Less than $569	90 (17.0)	58 (20.0)	64.4
$570 to $953	62 (11.7)	34 (11.7)	54.8
$954 to $1 476	64 (12.1)	33 (11.4)	51.6
$1 477 to $2 186	83 (15.8)	51 (17.6)	61.4
Greater to $2 186	231 (43.5)	114 (39.3)	49.4
Type of health system			
Public	225 (42)	125 (43.1)	55.6
Private	274 (52)	146 (50.3)	53.3
Other	32 (6)	19 (6.6)	59.4

### 3.2 Probit model analysis of fear

Several estimations were considered, including various explanatory variables such as the perception of government communication response, the perception of general government response, adaptability to remote work, age, gender, the perception that the socioeconomic impact is more important than health care, the perception of bad government response, the perception of own risk aversion, the existence of chronic diseases, family or relatives with COVID‐19 and education level. Table 2 presents the description of the statistically significant variables.

**Table 2:** Explanatory variable description

	I strongly disagree	I disagree	Not agree or disagree	I agree	I strongly agree
I consider myself a risk lover	210 (39.5)	204 (38.4)	68 (12.8)	38 (7.2)	11 (2.1)
Socioeconomic impact is more important than health care	236 (44.4)	197 (37.1)	64 (12.1)	22 (4.1)	12 (2.3)
Bad government response	110 (20.7)	165 (31.1)	61 (11.5)	85 (16.0)	110 (20.7)
Reduced economic activity	88 (20.7)	47 (8.9)	5 (0.6)	200 (37.7)	193 (36.4)
COVID‐19 knowledge level is good	90 (17.0)	53 (10.0)	41 (7.7)	200 (37.7)	147 (17.7)
I have a chronic sickness					
No	343 (64.6)				
Yes	188 (35.4)				
Family or relative with COVID‐19					
No	486 (91.5)				
Yes	45 (8.5)				

The result of the marginal probit model explaining the increase in fear is presented in Table 3. The estimation shows that fear has a positive relationship with variables such as existing chronic sickness, infected family, reduction in economic activity and perception of bad government response to the pandemic. In contrast, fear has a negative relationship with the knowledge about COVID‐19, education level and age. Moreover, those who regard the socioeconomic impact as less important than health care have less fear of contracting COVID‐19.

**Table 3:** Probit regression reporting marginal effect

Dependent variable Fears to get infected increased (Yes = 1, No = 0)	Marginal effect = dF/dx (robust standard error)	[95% confidence interval]
I have chronic sickness	0.1130542** (0.048302)	[0.0183, 0.2077]
Family or relative with COVID‐19	0.168712** (0.0764502)	[0.0187, 0.3186]
Reduce economic activity	0.0764951* (0.0228623)	[0.0317, 0.1213]
I consider myself a risk lover	‐0.0562568** (0.0240797)	[−0.1035, −0.0091]
COVID‐19 knowledge level is good	−0.0907256* (0.0238524)	[−0.1375, −0.0440]
Socioeconomic impact less important than health care	−0.059085** (0.0243702)	[−0.1069, −0.0113]
Age	−0.0310552*** (0.0179509)	[−0.0662, −0.0041]
Education	−0.056076** (0.02547)	[−0.1060, −0.0062]
Perception of bad government response	0.0565721* (0.0174914)	[0.0223, 0.0909]
Obs. P 0.551394 pred. P 0.5525956 (at x‐bar); Wald chi2(9) = 53.99 Prob >chi2 = 0.0000 Pseudo‐R2 = 0.0776 Log pseudolikelihood = −337.41454 N = 531		

* *P*‐value<0.10.

** *P*‐value <0.05.

*** *P*‐value <0.01.

## 4 DISCUSSION

Within the first one and a half months that the pandemic hit Chile, 54% of respondents proved to more fearful of contracting the disease. People who fear a COVID‐19 infection can exceed this percentage.

The increase in fear was higher in women (83.5%) than in men, in which it was 35%. Women in Chile may be more psychologically affected by the COVID‐19 pandemic^32^; that is, women may be more afraid of contracting the virus.

Our study corroborates other studies that mention that the worries and fear of getting COVID‐19 are influenced not only by the potential health impact but also socioeconomic effects and social vulnerability.^15^, ^30^, ^33^ Our study shows that the reduction in economic activity and perception of bad governmental response to the pandemic increases the fear. This result is consistent with Jakovljevic, Sugahara, Timofeyev and Rancic,^27^ with respect to government performance. Additionally, fear is less in people who think that the socioeconomic impact is less important than health care.

The existence of chronic sickness and family infectivity is also a predictor of fear in our study. This finding is consistent with other studies,^2^, ^29^, ^32^ which found that increased fear was related to perceived risk for loved ones and health anxiety, fear of becoming infected and fear of getting in contact with contaminated objects. The impact of having family members ill with symptomatic COVID‐19 affects the perception of the severity of the disease in individuals, generating greater fear of being infected due to its impacts not only on health but also on their socioeconomic environment.

We also found that fear is inversely proportional to age: it decreases when age increases. Despite adult people being reported to be at a higher risk, we found that they are less fearful of contracting the disease than young people.^34^ Some authors propose health literacy interventions in older people to promote healthy behaviours.^35^ However, we suggest that this attention to literacy should be given to young people because they tend to be more fearful than adult people. ^2^

Another relevant result of our study is that more educated and well‐informed people about COVID‐19 experience less fear. The result emphasizes the importance of education and information for public health policy. The government should make efforts to equip people with knowledge about COVID‐19. It should provide information on how to combat the virus to the less‐educated and young individuals.^16^

Given that the socioeconomic and health aspects help predict fears, governments must respond according to socioeconomic and health needs. They should also implement a good and credible communication system of the health situation and performance to combat the pandemic.^15^, ^23^, ^24^, ^29^, ^34^ Additionally, as mentioned by Ornell, Schuch, Sordi and Kessler,^36^ it is also necessary to implement public mental health policies. Anyway, the results about the variables explaining fear are determined by the temporal context of the pandemic; therefore, they could change over time.

## 5 CONCLUSIONS

The results of this cross‐sectional study suggest that people who are more fearful of contracting COVID‐19 are women, those with chronic illnesses, infectious families, those with loss of economic activity and those who perceive a bad governmental response to the pandemic. In comparison, fear decreased with the knowledge about the pandemic, education level and age. Therefore, governments should prioritize these variables: health, social and economic policy implementation.

Some limitations of our study are that our sample includes a high proportion of people with relatively high educational levels, and the convenience sampling system. The online data collection method limits the generalizability of the results. Another aspect to consider is that the results about the variables explaining fear are determined by the temporal context of the pandemic; therefore, they could change over time. Therefore, studying the determinants of temporal changes in the fears faced by individuals due to the pandemic is an interesting line of research to develop.

## CONFLICT OF INTEREST

LG and AC declare they have no conflicts of interest that are relevant to this manuscript.

## AUTHOR CONTRIBUTIONS

AC conceptualized the design, performed formal analysis and investigation, contributed to resources, and wrote the original draft. LG modelled estimation, and wrote, reviewed and edited the manuscript.

## Data Availability

The data sets that support this study's findings are available from the corresponding author upon reasonable request.
